# Development of a clickable bimodal fluorescent/PET probe for in vivo imaging

**DOI:** 10.1186/s13550-015-0120-4

**Published:** 2015-08-19

**Authors:** Andreas Paulus, Pooja Desai, Brandon Carney, Giuseppe Carlucci, Thomas Reiner, Christian Brand, Wolfgang A Weber

**Affiliations:** Department of Radiology, Memorial Sloan Kettering Cancer Center, 1275 York Avenue, 10065 New York, NY USA; Department of Chemistry and Biochemistry, Hunter College of the City University of New York, 10065 New York, NY USA; Program in Chemistry, The Graduate Center of the City University of New York, 10018 New York, NY USA; Weill Cornell Medical College, 10065 New York, NY USA; Molecular Pharmacology & Chemistry Program, Memorial Sloan Kettering Cancer Center, New York, NY USA

## Abstract

**Background:**

Fluorescent imaging agents are becoming evermore important in preclinical and clinical research. They do, however, suffer from poor tissue penetration, which makes optical fluorescence imaging incompatible with whole-body imaging techniques. The design of novel bimodal PET active and fluorescent tracers could therefore combine the benefits of optical imaging with radioactively labeled imaging probes. Herein, we report the synthesis and evaluation of a clickable ^18^F-labeled fluorescent dye.

**Methods:**

An azide-modified BODIPY-Fl dye could be successfully radio-labeled with ^18^F using an ^18^F/^19^F exchange reaction of the boron-fluoride core of the BODIPY dye to yield a clickable bimodal PET/fluorescent imaging tool. In vitro as well as in vivo imaging (PET/fluorescence) using a bombesin analog was conducted to study the applicability of the dual-modality imaging probe.

**Results:**

We use the radio-labeled small molecule, ^18^F-BODIPY-azide to label site-specifically different targeted peptides, based on a standard modular labeling protocol. Following the synthesis of a bimodal bombesin analog, we determine the peptide tracer’s performance in vitro and in vivo, exploring both the optical as well as PET imaging capabilities.

**Conclusion:**

This versatile methodology has the potential to have a transformational impact on ^18^F radiotracer synthesis, opening the door for rapid screening of novel-labeled peptide tracers, both on the cellular (optical) as well as whole-body (PET) level.

**Electronic supplementary material:**

The online version of this article (doi:10.1186/s13550-015-0120-4) contains supplementary material, which is available to authorized users.

## Background

Positron emission tomography (PET) allows non-invasive whole-body imaging by detecting pairs of gamma rays, a secondary product of positron decay of radionuclides. Among the commonly used radionuclides for PET, ^18^F is often the radioelement of choice due to its relatively short half-life and multifaceted chemistry. However, the spatial resolution of PET is limited and cannot detect microscopic lesions or resolve the heterogeneity of tissues at the cellular level [1]. In contrast, optical fluorescence imaging has very high spatial resolution, making it a valuable technique for the development of intraoperative imaging agents [2–4]. This technology, however, suffers from low penetration depths, making its strengths and weaknesses orthogonal to PET.

Therefore, dual-modality imaging probes have the potential to combine the advantages of both imaging techniques and allow imaging from the whole-body down to the cellular level [5]. Two major challenges in the development of dual-modality imaging agents are the usually increased synthetic complexity and the effect of the fluorescent dye on the biodistribution of the imaging agent. In order to reduce the steric impact resulting from the conjugation of a bimodal imaging tag to a small peptide ligand, we got interested in ^18^F/^19^F exchange reactions used to transform fluorescent dyes into dual-modality PET/fluorescent imaging dyes [6–10]. The advantage of this approach is that a targeting molecule is only modified at a single site, allowing both optical and PET labeling without increased steric demand, lowering the chances of compromising targeting efficiency. In comparison to previous reports, we wanted to go a step further developing a dual-modality linker—using the previously described ^18^F/^19^F exchange reaction with the BODIPY-Fl dye—which can be site-specifically conjugated to biomarkers in the second step.

Herein, we describe a synthetic method for labeling peptides with a modular ^18^F/^19^F-labeling protocol, allowing the fast and rapid design of multiple optical/PET labeled peptides with just one radio-labeled synthetic intermediate. Specifically, our approach is based on a ^18^F/^19^F exchange reaction of a BODIPY-Fl dye which is then coupled to peptides by a click reaction. We use four different targeted peptides to show the general applicability of our approach, and test one bimodal bombesin receptor targeting peptide in a PC-3 prostate cancer model, both in vitro as well as in vivo.

## Methods

### Materials

Commercially available compounds were used without further purification unless otherwise stated. Alkyne-modified bombesin analog (RM2-alkyne, BBN) (Leu-Sta-His-Gly-Val-Ala-Trp-Gln-D-Phe-4-amino-1-carboxymethyl-piperidine-4-pentynoic acid) was synthesized with a peptide synthesizer (Rainin/Protein Technologies) and purified by high performance liquid chromatography (HPLC) using a reversed phase XTerra® preparative column (C18, 10 μm, 19 mm × 250 mm). Alkyne-modified exendin-4 (E4_x12_), CCK2, and pHLIP were purchased from C S Bio Co. (Menlo Park, CA). [^125^I-Tyr^4^]-bombesin was purchased from Perkin Elmer (Boston, MA). BODIPY-Fl *N*-hydroxysuccinimidyl (NHS) ester was purchased from Life Technologies (Carlsbad, CA). Phosphate buffered saline (PBS) and Dulbecco’s Modified Eagle Medium (DMEM) were purchased from the media preparation facility at Memorial Sloan Kettering Cancer Center (New York, NY, USA). PC-3, a human prostate cancer cell line was purchased from ATCC (Manassas, VA). All HPLC purifications (1.0 mL/min, buffer A; 0.1 % TFA in water, buffer B; 0.1 % TFA in CH_3_CN) were performed on a Shimadzu UFLC HPLC system equipped with a DGU-20A degasser, a SPD-M20A UV detector, a LC-20AB pump system, a CBM-20A communication BUS module, a FRC-10A fraction collector, and a Scan-RAM radio-TLC/HPLC-detector from LabLogic using a reversed phase Atlantis® T3 column (C18, 5 μm, 4.6 mm × 250 mm). Quality controls of radio-labeled compounds were analyzed using a Shimadzu HPLC system with a Posi-Ram detector. Electrospray ionization mass spectrometry (ESI-MS) spectra were recorded with a Waters Acquity UPLC (Milford, CA) with electrospray ionization SQ detector. High-resolution mass spectrometry (HRMS) spectra were recorded with a Waters LCT Premier system (ESI). The radioactivity of the binding assay was counted with a WIZARD^2^ automatic γ-counter from Perkin Elmer (Boston, MA). Nunc™ Lab-Tek™ II Chamber Slide™ Systems were analyzed by an inverted confocal microscope (Leica TCS SP5 II) using an objective with ×20 magnification. MicroSuite FIVE software was used to register images, and Fiji software was used to manually adjust and analyze images. Small animal PET imaging data were recorded on an Inveon® PET/CT from Siemens (Knoxville, TN) and epifluorescence imaging was conducted with an IVIS spectrum fluorescence imaging system (PerkinElmer).

### Preparation of BODIPY-Fl-PEG4-Azide 1

A solution of 11-Azido-3,6,9-trioxaundecan-1-amine (2.2 mg, 10 μmol, 1.1 eq.) in anhydrous dimethylformamide (20 μL) was added slowly to a stirred solution of BODIPY-Fl NHS ester (2.0 mg, 5.1 μmol, 1.0 eq.) and *N*,*N*-Diisopropylethylamine (2.6 mg, 20 μmol, 0.8 eq.) in anhydrous dimethylformamide (800 μL) at room temperature and the resulting mixture was stirred in the dark at room temperature for 2 h. Addition of water (800 μL) and purification by HPLC (1 mL/min, 5 to 95 % B in 20 min) afforded **1** (2.5 mg, 99 %) as a red solid; *t*_R_ = 14.3 min. ESI-MS(+): *m/z* (%) = 515.3 (100) [M + Na]^+^.

### Preparation of ^18^F-BODIPY-azide 2

The radiolabeling of the BODIPY dye was performed according to the isotope exchange method reported previously [6]. Briefly, the activated aqueous ^18^F solution is transferred into a drying vessel containing tetra-*n*-butylammonium hydrogen carbonate (80 μL) as a phase transfer agent. Acetonitrile (3 × 3.0 mL) was added and the solution of ^18^F was dried by heating to 100 °C with a continuous flow of argon. After reconstitution of ^18^F (44 ± 5 mCi) in anhydrous acetonitrile (100 μL), a solution of BODIPY-azide **1** (50 μg, 0.1 μmol) and SnCl_4_ (0.1 M in acetonitrile, 100 μL) was added to the solution with the activity and the reaction solution was stirred at 37 °C for 30 min. After addition of water (400 μL), purification by HPLC (0.8 mL/min, 30 to 95 % B in 30 min) afforded **2** (RCY: 3.8 ± 1.4 %) with a specific activity of (5 ± 0.5 mCi/μmol).

### Preparation of ^18^ F-BODIPY-BBN 3

To a solution of ^18^ F-BODIPY-azide **2** (700 ± 300 μCi), BODIPY-azide **1** (50 μg, 0.10 μmol) and alkyne-modified BBN **4** (0.16 mg, 0.10 μmol) in PBS (250 μL) was added a premixed solution of CuSO_4_ (100 μg, 0.63 μmol) and ascorbic acid (550 μg, 3.1 μmol) in PBS (63 μL). The reaction solution was stirred in the dark at room temperature for 1 h. Addition of acetonitrile (150 μL) and purification by HPLC (0.8 mL/min, 5 to 95 % B in 15.9 min) afforded **3** (decay-corrected RCY: 45 ± 4 %) with an estimated specific activity of (625 ± 100 μCi/μmol) and a radiochemical purity of >95 %.

### Preparation of BODIPY-BBN 5

To a solution of BODIPY-azide **1** (0.4 mg, 0.8 μmol, 1.0 eq.) and alkyne-modified bombesin **4** (1.1 mg, 0.8 μmol, 1 eq.) in PBS (0.5 mL) was added a solution of CuSO_4_ (0.8 mg, 5.0 μmol, 6.0 eq.) and ascorbic acid (4.4 mg, 25 μmol, 31 eq.) in PBS (0.5 mL) and the resulting mixture was stirred in the dark at room temperature for 1 h. Addition of acetonitrile (200 μL) and purification by HPLC (1 mL/min, 5 % to 95 % B in 20 min) afforded **5** (0.4 mg, 27 %) as a red solid: t_R_ = 11.1 min. ESI-MS(+): *m/z* (%) = 914.0 (100) [M + 2H]^2+^. HRMS (ESI): *m/z* calcd for C_89_H_128_BF_2_N_22_O_17_: 1826.9889; found: 1826.9968 [M + H]^+^.

### Preparation of ^18^F-BODIPY-labeled peptides

Using the previous described click chemistry approach, we attached successfully ^18^F-BODIPY-azide **2** to various peptides, such as exendin-4, pHLIP, and CCK2 and obtained three different peptidic dual-modality imaging agents: Analytical data for ^18^F-BODIPY-CCK2: *t*_r_ = 14.7 min; ESI-MS(+): *m/z* (%) = 1134.2 (100) [M + Na + H]^2+^. Analytical data for ^18^F-BODIPY-pHLIP: *t*_r_ = 12.8 min; ESI-MS(+): *m/z* (%) = 1168.7 (100) [M + Na + 3H]^4+^, 1557.8 (40) [M + 2H + Na]^3^. Analytical data for ^18^F-BODIPY-exendin-4: *t*_r_ = 13.2 min; ESI-MS(+): *m/z* (%) = 930.9 (35) [M + 5H]^+^, 1157.8 (100) [M + 4H]^4+^, 1550.1 (30) [M + 3H]^3+^.

### Cell culture

The human prostate cancer cell line PC-3, a G-protein coupled receptor-positive cell line was used for in vitro receptor-binding studies at passages 3–8. Cell culture was performed as previously described [11]. The cells were grown in F-12 K medium containing 2 mM L-glutamine and 1500 mg/L sodium bicarbonate and passaged regularly at 70–80 % confluence every 3–4 days. PC-3 cells were cultured according to the recommendations of American Type Culture Collection and Caliper Life Sciences under 37 °C with 5 % CO_2_.

### Animals

All mouse model experiments and procedures were performed in accordance with the guidelines set by the Institutional Animal Care and Use Committee at Memorial Sloan Kettering Cancer Center. Male athymic nude mice (Charles River Lab; Cr1:Nu-Foxn1nu, 6–8 weeks, 20–25 g) were used for subcutaneous mouse model. PC-3 cells (5.0 × 10^6^) were suspended in media (150 μL), mice were anesthetized with 2 % isoflurane gas in 2 L/min medical air, and PC-3 cells were injected subcutaneously in the right shoulder to establish tumor-bearing mice (4–8 mm tumor diameter) after 2 weeks. For intravenous injections, mice were gently heated with a heating lamp, placed on an injection restrainer, tail cleaned with sterilized alcohol pads, and the imaging agent was injected to the lateral tail vain.

### In vitro cell imaging

To determine BODIPY-BBN uptake in vitro, PC-3 cells (1.0 × 10^4^ per well) were seeded in a two-well chamber slide (Nunc™ Lab-Tek™ II Chamber Slide™ System) containing growth media (1 mL) and incubated at 37 °C for 24 h. To study the uptake of BODIPY-BBN, PC-3 cells were incubated with **5** (10 nM in PBS, 37 °C for 30 min). At the same time blocking studies were performed. PC-3 cells were pre-incubated with RM2 (1000 nM in PBS, 37 °C for 5 min) before incubation with BODIPY-BBN (10 nM in PBS, 37 °C for 30 min). After washing with media (1.0 mL), blue whole cell stain was added and incubated at room temperature for 5 min. The cells were washed with PBS (3 × 1.0 mL), living cells were imaged using an inverted confocal microscope (Leica TCS SP5 II), and data were analyzed with Fiji.

### In vitro receptor-binding assay

A competitive receptor-binding assay with [^125^I-Tyr^4^]-bombesin was performed to determine the receptor-binding affinity of BODIPY-BBN **5**. PC-3 cells (5.0 × 10^5^ cells per vial) were added to vials containing medium (900 μL). For the assay, equal volumes (50 μL) of [^125^I-Tyr^4^]-bombesin and BODIPY-BBN **5** were added. The concentration of [^125^I-Tyr^4^]-bombesin was 10^−12^ M. Increasing concentrations (10^−6^ to 10^-14^) of BODIPY-BBN **5** were added. After agitation at room temperature for 2 h, the cells were harvested by a filter system. The filter for each vial was collected and counted in a γ-counter. Experiments as well as each data point were conducted in triplicate. Data were analyzed with GraphPad Prism 6 Software to determine the IC_50_ value of BODIPY-BBN **5**.

### Surface fluorescence imaging

With the identified in vitro binding affinity of BODIPY-BBN **5** to GRP receptor-positive PC-3 cells, surface fluorescence imaging experiments with an IVIS spectrum fluorescence imaging system (PerkinElmer) were conducted with subcutaneous tumor-bearing mice. Mice were injected intravenously with the imaging agent BODIPY-BBN **5** (33 μg, 18 nmol) in PBS (5 % DMSO, 200 μL), sacrificed after 1 h post injection, and then ex vivo tumor as well as muscle epifluorescence were quantified using Living Image® 4.3 software (Caliper Life Sciences). For a control experiment, mice were injected with PBS (200 μL) and organs of interest were harvested for ex vivo optical imaging.

### PET/CT imaging

Small animal PET/CT imaging was performed on an Inveon® multi-modality PET/CT imaging scanner (Siemens). ^18^F-BODIPY-BBN **3** (55 ± 10 μCi) in PBS (4 % DMSO, 200 μL) was injected into tumor-bearing nude mice (*n* = 3) via tail vain. At 30 min after injection, the mice were anesthetized with 1.5-2.0 % isoflurane (Baxter Healthcare) at 2 mL/min in oxygen and dynamic PET/CT imaging was accomplished over 90 min. Images were analyzed using AsiPro VM™ software (Concorde Microsystems) and Inveon research workplace 4.1 software (Siemens Healthcare). After PET imaging, mice were sacrificed by asphyxiation with CO_2_ and tumor as well as muscle were harvested, weighed, and counted using a WIZARD^2^ automatic γ-counter from Perkin Elmer. The percentage of tracer uptake stated as percentage injected dose per gram of tissue (%ID/g) was calculated as the activity bound to tissue per organ weight per actual injected dose, decay-corrected to the start time of counting.

## Results

**Fig. 1:**
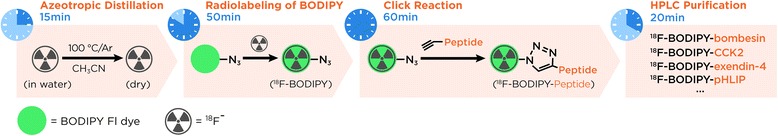
Conceptual design of the synthesis and application of a bimodal click chemistry tool. After azeotropic distillation of [^18^ F]-F^−^ n.c.a., the radionuclide was reacted with the BF_2_ group of an azide-modified BODIPY-FL dye, yielding a ^18^ F-labeled, bimodal imaging agent. A modular Cu-click protocol allowed the rapid conjugation of the azide dye to targeted peptides, quickly yielding a variety of compounds with only little synthetic effort

Our concept for the synthesis of bimodal imaging agents is shown in Fig. 1. First, the azeotropic distillation of an aqueous fluorine-18 solution allows us to radiolabel an azide-modified BODIPY-FL to yield a dual-modality (radioactive/fluorescent) imaging agent. The clickable bimodal imaging tracer is then conjugated in a bioorthogonal cycloaddition reaction to various peptides, yielding dual-modality imaging agents quickly via a modular synthetic protocol.

Synthesis of our ^19^F-labeled fluorescent analog BODIPY-azide **1** started from commercial available BODIPY-Fl NHS ester (Additional file 1: Figure S1A). The commercially available amine reactive ester was reacted with 11-Azido-3,6,9-trioxaundecan-1-amine and BODIPY-azide **1** was isolated in 99 % yield after HPLC purification. The bombesin analog RM2 (BBN) [12] was synthesized manually according to standard Fmoc chemistry [13] and modified on the N-terminus with the unnatural amino acid (S)-2-Amino-4-pentynoic acid with a yield of 71 % (see Additional file 1: Supporting information). Finally, bioorthogonal 1,3-dipolar cycloaddition of BODIPY-azide **1** to alkyne-modified BBN **4** afforded the BODIPY-BBN **5** in 34 % yield (Additional file 1: Figure S2).

**Fig. 2:**
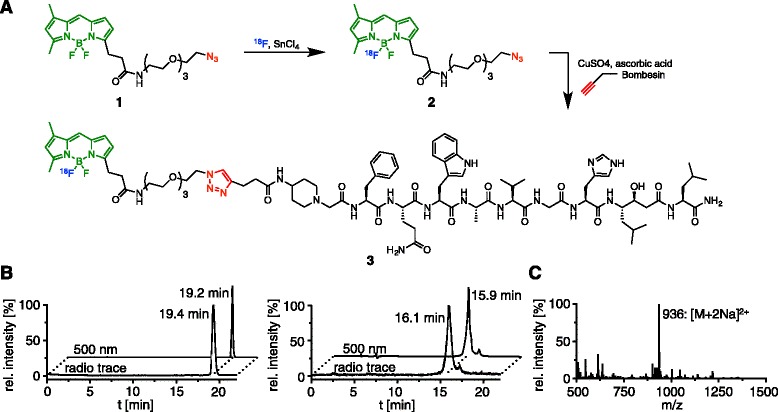
Synthesis and analytical data of ^18^F-BODIPY-BBN **3**. **a** Tin tetrachloride-catalyzed ^18^F-radiolabeling of BODIPY-Fl azide **1** followed by CuAAC click chemistry with alkyne-modified bombesin analog (RM2-alkyne) to yield ^18^F-BODIPY-BBN **3**. **b** HPLC chromatograms (radio trace and absorbance at 500 nm) of ^18^F-BODIPY-N_3_ **2** (*left*) and ^18^F-BODIPY-BBN **3** (*right*). **c** Electrospray ionization mass spectrometry of cold BODIPY-BBN

The radio-labeled dual-modality imaging agent ^18^F-BODIPY-BBN **3** was synthesized by a two-step procedure, similar to previous reports (Fig. 2a) [6, 8, 10]. First, BODIPY-azide **1** was radio-labeled with ^18^F using the strong Lewis acid SnCl_4_ in dry acetonitrile. After 30 min of incubation, ^18^F-BODIPY-azide **2** was isolated by HPLC purification in 3.8 ± 1.4 % radiochemical yield (Fig. 2b). The resulting dual-modality imaging tool was subjected to standard azide-alkyne cycloaddition (CuAAC) reaction conditions (alkyne-modified BBN **4**, ascorbic acid, copper sulfate, and room temperature), yielding the peptide-based dual-modality imaging agent. After HPLC purification, the decay-corrected radiochemical yield for the second synthetic step was determined to be 45 ± 4 %, with a radiochemical purity of >95 %, and a specific activity of 625 μCi/μmol (Fig. 2b). BODIPY-azide 1 (50 μg) was added to the reaction mixture to accelerate the 1,3-dipolar azide-alkyne cycloaddition. The synthetic preparation of the dual-modality peptide-based imaging agent was achieved in 2.5 h (including radiolabeling, HPLC purification of **2**, evaporation of solvents, copper-catalyzed [3 + 2] Huisgen cycloaddition, HPLC purification of **3** and formulation of an injectable solution).

**Fig. 3:**
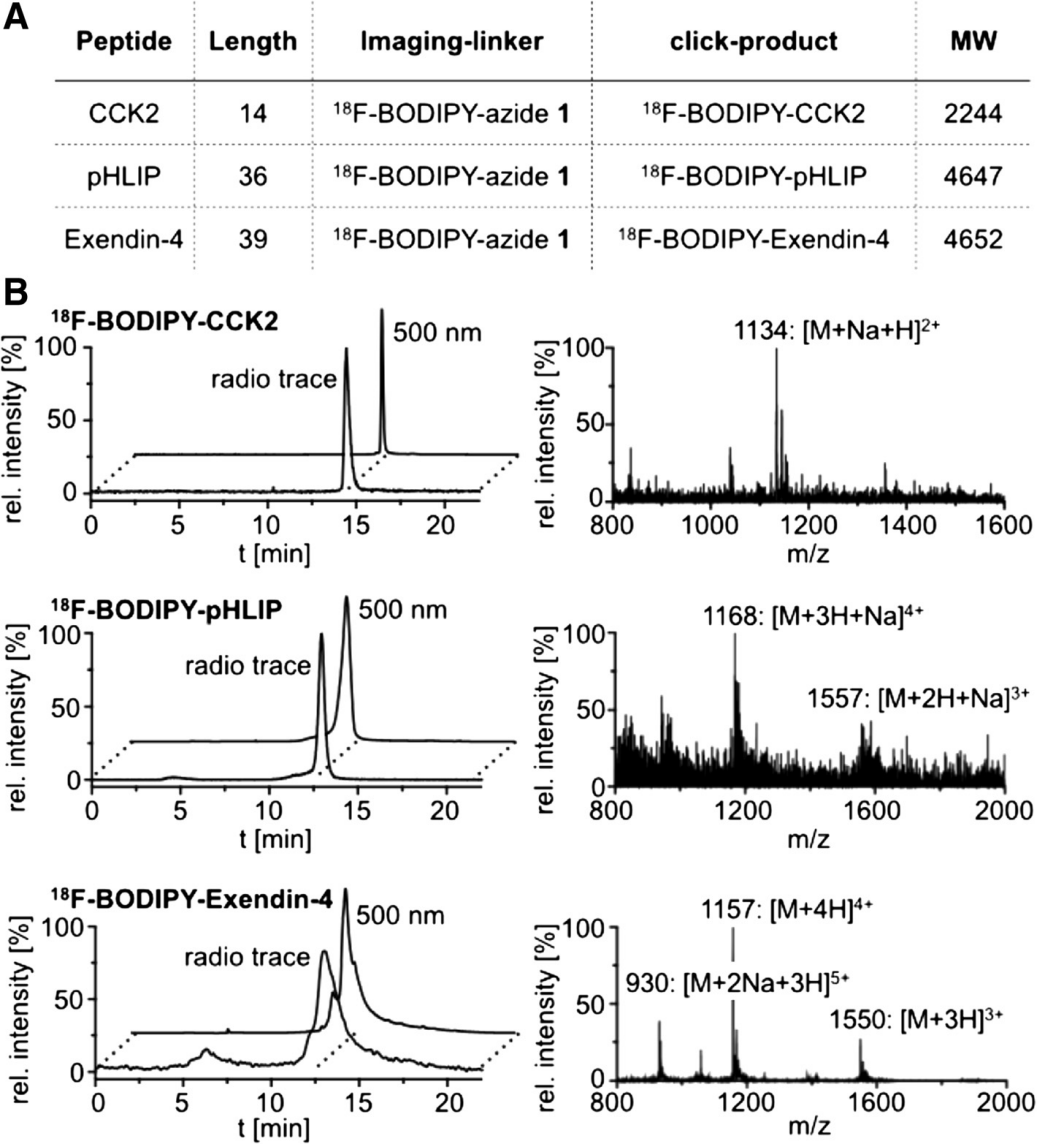
Application of the bimodal click chemistry tool for the synthesis of dual-modality peptidic imaging agents. **a** Overview of three alkyne-modified peptides and the click products with their molecular weights. **b** HPLC chromatograms and ESI-MS data of the synthesized dual-modality peptide-based imaging agents

To show the general applicability of this new approach, ^18^F-BODIPY-azide **2** was coupled to three other peptides, which were previously described for imaging of tumors by using either PET imaging or optical imaging (Fig. 3a) [5, 14–16]. Using standard click reaction conditions, we successfully synthesized three different dual-modality imaging agents with the same precursor (Fig. 3b). For hydrophobic peptides (exendin-4 and pHLIP), dimethylsulfoxide can be added to the reaction solution to increase peptide solubility. HPLC ESI-MS confirmed that the identity of the products where the ionized masses found in the ESI-MS spectra corresponded with the calculated molecular weight of the click product.

**Fig. 4:**
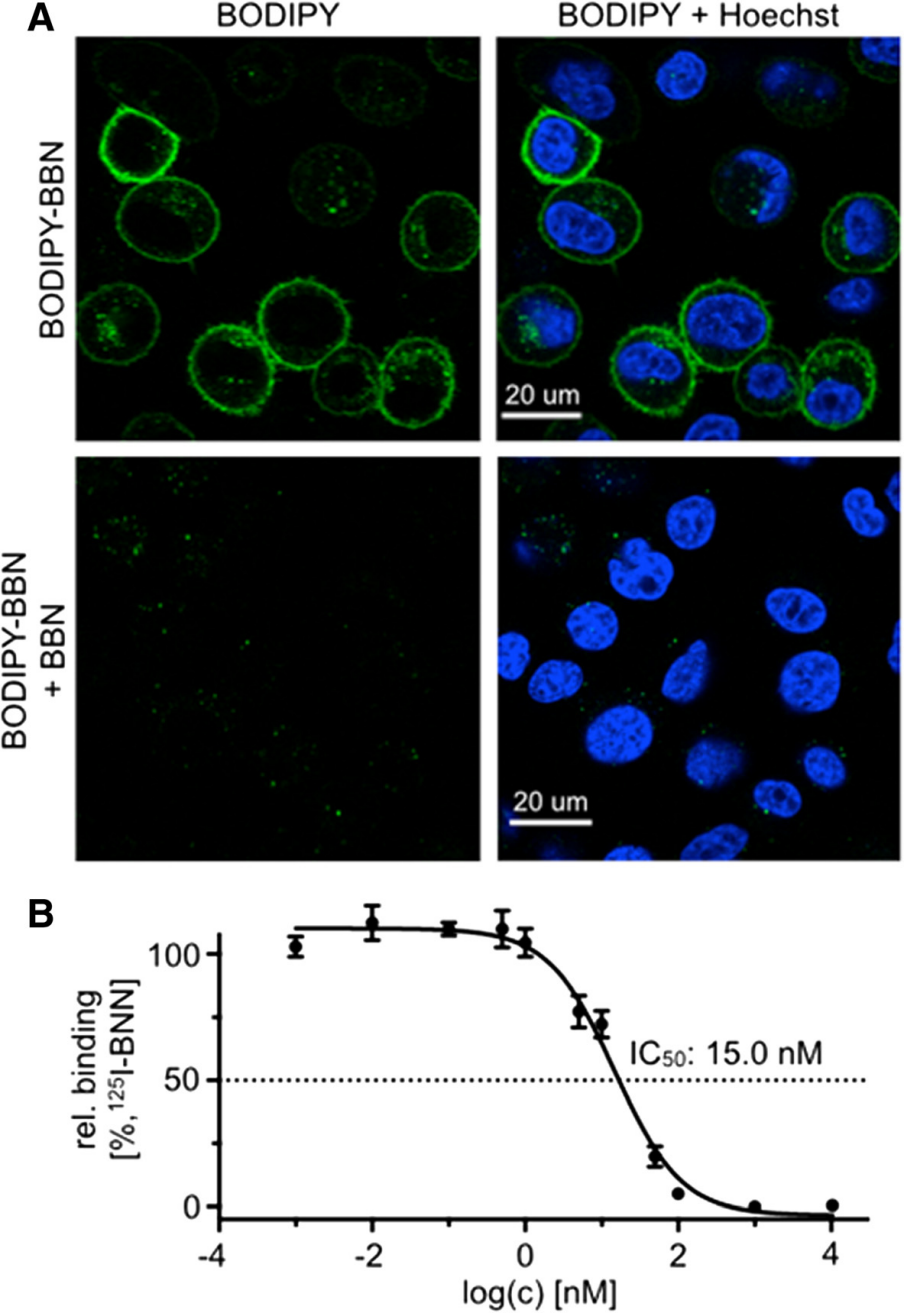
In vitro uptake and inhibition studies of BODIPY-BBN **5** using GRP receptor overexpressing PC-3 cells. **a** Confocal microscopy imaging experiment demonstrated cell surface binding of the green fluorescent imaging agent in the green channel (BODIPY, *top row*); Nuclei were counterstained with Hoechst dye (*blue*, *top row*); Blocking studies of BODIPY-BBN **5** were performed with a 100-fold excess of unlabeled GRP receptor antagonist [D-Phe^6^]-BN(6–13)-ethylester (*bottom row*). **b** IC_50_ values of **5** were evaluated by a competitive binding assay using [^125^I-Tyr^4^]-bombesin

The binding of BODIPY-BBN **5** toward GRP receptor overexpressing PC-3 cells was evaluated in confocal live-cell imaging (Fig. 4a). After incubation of BODIPY-BBN **5** (10 nM, 30 min), cells were treated with blue whole cell stain (5 min) and washed with PBS. Confocal microscopy demonstrated antagonist-specific binding of BODIPY-BBN **5** (green, top row) to the membrane surface [12]. To show GRP receptor specificity of BODIPY-BBN, PC-3 cells were pre-incubated with an excess of unmodified GRP receptor antagonist [D-Phe^6^]-BN(6–13)-ethylester which showed complete suppression of green fluorescent signal (bottom row). We also confirmed the binding affinity of BODIPY-BBN **5** using [^125^I-Tyr^4^]bombesin in a competitive binding assay (Fig. 4b). Compared to an earlier reported IC_50_ of RM2 (7.7 ± 3.3 nM) [12] we observed a slightly higher IC_50_ of 15 ± 3.5 nM for our bimodal imaging agent BODIPY-BBN **5**. We then determined the stability of our imaging tracer in human serum (37 °C) at pre-determined time points showing >90 % intact fluorescent imaging agent after 60 min of incubation (Additional file 1: Figure S6).

**Fig. 5:**
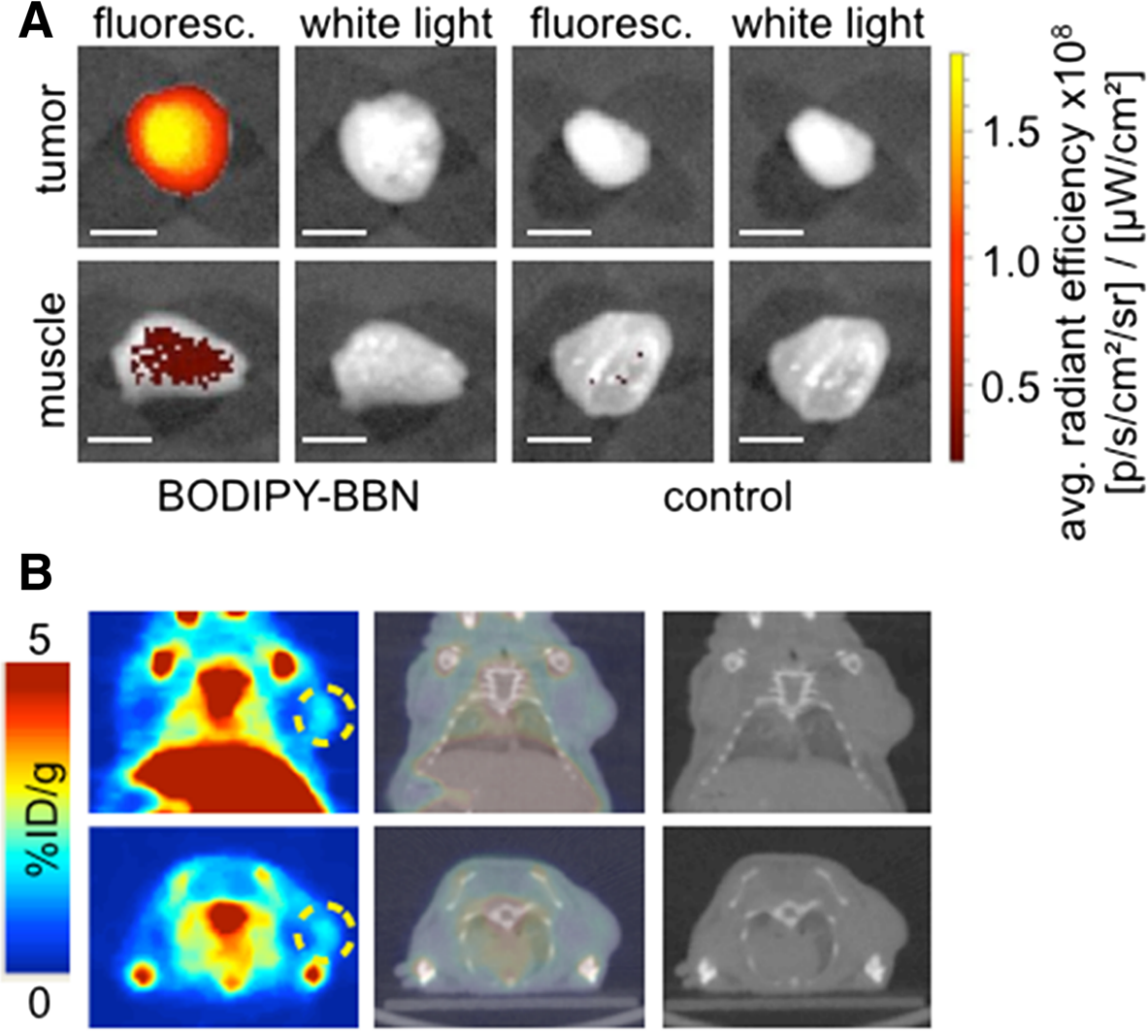
Visualization of PC-3 tumors by optical imaging as well as PET imaging using the dual-modality imaging agent ^18^F-BODIPY-BBN. **a** Ex vivo fluorescent imaging of tumor and muscle after intravenous injection of BODIPY-BBN **5** (18 nmol) in PBS (4 % DMSO, 200 μL); For a control experiment PC-3 tumor-bearing mice were injected with PBS (4 % DMSO, 200 μL); scale bar: 5 mm. **b** In vivo small animal PET/CT images (30 min) after intravenous injection of ^18^F-BODIPY-BBN **3** (55 ± 10 μCi) in PBS (4 % DMSO, 200 μL)

After evaluating BODIPY-BBN **5** as a successful imaging agent in vitro, we explored the dual-modality imaging properties (optical imaging as well as PET imaging) for GRP receptor overexpressed tumors using a PC-3 subcutaneous tumor mouse model (Fig. 5). After 1 h post intravenous injection of BODIPY-BBN **5**, ex vivo fluorescent imaging of tumor and muscle revealed uptake of the imaging agent in the tumor with GRP receptor overexpression (Fig. 5a). Analog to this finding, the intensity of specific fluorescent signal of the imaging agent yielded a tumor to muscle ratio of 5.7 (Additional file 1: Figure S4). Control mice, injected with PBS (*n* = 3), did not show fluorescence in their tumor tissue, yielding a tumor to muscle ratio of 0.21.

We injected tumor-bearing mice (PC-3, *n* = 3) with ^18^F-BODIPY-BBN **3** to determine the potential of the probe as a whole-body imaging agent (Fig. 5b). Although unexpected high liver and bone uptake were observed, we could clearly visualize the tumor with 1.2 ± 0.2 percent injected dose per gram (%ID/g) in PET/CT images 30 min post intravenous injection of ^18^F-BODIPY-BBN **3** (55 ± 10 μCi). After PET/CT imaging, mice were sacrificed, organs of interest (tumor as well as muscle) were harvested, and the specific uptake of tracer was determined by gamma-counting. Mirroring the optical imaging, we observed a tumor to background ratio of 5.2 (Additional file 1: Figure S4).

## Discussion

Positron emission tomography is a well-established diagnostic tool in modern medicine, particularly in oncology. During the last decade, tremendous success has been achieved in the development of novel peptide-based targeted imaging agents [17, 18]. Using a radio-labeled peptide-based imaging probe, PET can be used for the visualization as well as the quantification of cancer cell-specific protein overexpression in tumor tissue [19]. However, PET imaging has its limitation in spatial resolution [1]. A dual-modality imaging probe that is both radioactive as well as fluorescent can overcome this limitation and allows imaging from the whole-body to the cellular level.

Over the last years, the development of dual-modality imaging platforms—combining PET imaging and optical imaging—has evolved into a fast growing research field [20, 21]. Several reports also describe the synthesis of dual-modality peptide-based imaging agents [4, 22, 23]. In 2009, Kimura et al. developed a cross-linking strategy for stoichiometric coupling of DOTA for radiolabeling with ^64^Cu as well as near-infrared dye Cy5.5 for optical imaging to a disulfide knottin peptide [23]. They, however, observed higher kidney and less tumor uptake of the dual-modality integrin receptor imaging agent, presumably due to the bulkiness of the conjugated imaging tag. In 2014, our group presented a linear approach for the synthesis of a dual-modality imaging agent to reduce the impact of the bimodal imaging tag [5]. Here, the conjugation of the bimodal tag containing the fluorescent dye, together with the hexaamine copper chelator DiAmSar, resulted in a decreased binding affinity of the dual-modality imaging tracer compared to just a PET [24] or a fluorescent [25–27] exendin-4-based imaging agent.

In order to avoid an impact on target binding and biodistribution, the design of a dual-modality imaging agent should ideally aim for the incorporation of a fluorochrome as well as radionuclide with a small “footprint”. We therefore developed a clickable ^18^F-BODIPY-azide bimodal imaging tool for site-specific conjugation to biomarkers for in vivo imaging. This approach is based on several previous studies which have indicated the feasibility of using BODIPY dyes as bimodal imaging agents bimodal [6–8]. The principal advantage of this approach is that the chemical properties of the optical and bimodal optical/nuclear imaging agent are the same (Fig. 1). However, it is to mention that labeling first and then clicking the dual-modality imaging tracer to a peptide in general leads to lower specific activities than some other labeling approaches.

Keliher et al. recently showed that the labeling efficiency is strongly dependent on the type of BODIPY dye [8]. Their conversion after 30 min incubation reaches from 16 to 90 % with the same labeling procedure for different dyes. Similar results were obtained by Liu et al. [6] and Hendricks et al. [7]. Hendricks also observed high in vivo stability, which was shown by injecting a non-targeted ^18^F-BODIPY small molecule, demonstrating no release of radioactive fluorine. In comparison to these previously reported studies, our in vivo experiments resulted in a much higher uptake of radioactivity in bone, which likely originates from free ^18^F. We assume that the accumulation of activity in the bones is based on insufficient metabolic stability of the borane-fluorine bond of ^18^F-BODIPY-BBN **3**. One reason for the decreased in vivo stability of our probe, compared to earlier examples [6–8], might be that different BODIPY core structures have different metabolic half lifes.

## Conclusions

In summary, we investigated the synthesis and application of a clickable bimodal fluorescent/PET imaging tool. With the successful incorporation of ^18^F into a clickable BODIPY-Fl linker, we were able to site-specifically conjugate the dual-modality imaging tracer to four different peptides. We then investigated the properties of ^18^F-BODIPY-BBN as a GRP receptor targeting imaging agent in vitro as well as in vivo. Even though we could visualize a tumor in PC-3 xenograft mouse model by ex vivo surface fluorescent imaging as well as whole-body PET imaging, further studies with different—and potentially more stable—BODIPY dyes will be conducted to reduce the off target bone uptake.

## Additional file

Additional file 1: Supplementary data on the synthesis of BODIPY-BBN, its in-vitro characterization and its in-vivo biodistribution and stability. Figure S1. Synthesis and analytical data of BODIPY-azide **1**. **Figure S2.** Synthesis of BODIPY-BBN **5** using CuAAC click chemistry. **Figure S3.** In vitro cell binding study of BODIPY-BBN **5** using GRPr overexpressing PC-3 cells. **Figure S4.** Quantification of tumor uptake comparing fluorescent imaging as well as gamma-counting. **Figure S5.** In vivo small animal PET/CT images (60 min) of PC-3 tumor-bearing nude mice after intravenous injection of ^18^F-BODIPY-BBN **3** (55 ± 10 μCi) in PBS (4 % DMSO, 200 μL). **Figure S6.** Percent intact fluorescent ^19^F-BODIPY-BBN in human serum at different time points (0, 15, 30, 45, and 60 min) after incubation at 37 °C.
