# Supplementary material for: Development of a clickable bimodal fluorescent/PET probe for in vivo imaging
Source: EJNMMI Res. 2015 Aug 19;5:43. doi: 10.1186/s13550-015-0120-4 (PMC4540712; doi:10.1186/s13550-015-0120-4)
Supplement: Additional file 1: Supplementary data on the synthesis of BODIPY-BBN, its in-vitro characterization and its in-vivo biodistribution and stability. Figure S1. — Synthesis and analytical data of BODIPY-azide 1. Figure S2. Synthesis of BODIPY-BBN 5 using CuAAC click chemistry. Figure S3. In vitro cell binding study of BODIPY-BBN 5 using GRPr overexpressing PC-3 cells. Figure S4. Quantification of tumor uptake comparing fluorescent imaging as well as gamma-counting. Figure S5. In vivo small animal PET/CT images (60 min) of PC-3 tumor-bearing nude mice after intravenous injection of 18F-BODIPY-BBN 3 (55 ± 10 μCi) in PBS (4 % DMSO, 200 μL). Figure S6. Percent intact fluorescent 19F-BODIPY-BBN in human serum at different time points (0, 15, 30, 45, and 60 min) after incubation at 37 °C. [file 13550_2015_120_MOESM1_ESM.docx]

**Development of a clickable bimodal fluorescent/PET probe for in vivo imaging**

- Supporting Information -

Andreas Paulus,^1,#^ Pooja Desai,^1^ Brandon Carney,^1,2,3^ Giuseppe Carlucci,^1^ Thomas Reiner,^1,4^ Christian Brand,^1,#^ Wolfgang A. Weber*^,1,5^

^1^ Department of Radiology, Memorial Sloan Kettering Cancer Center, New York, NY, 10065, USA.

^2^ Department of Chemistry and Biochemistry, Hunter College of the City University of New York, New York, NY, 10065, USA.

^3^ Ph.D. Program in Chemistry, The Graduate Center of the City University of New York, New York, NY, 10018, USA.

^4^ Weill Cornell Medical College, New York, NY, 10065, USA.

^5^ Molecular Pharmacology & Chemistry Program, Memorial Sloan Kettering Cancer Center, New York, NY, USA.

*^#^* These authors contributed equally.

*W. A. Weber, MD

Department of Radiology, Memorial Sloan Kettering Cancer Center, 1275 York Avenue, New York, NY 10065.

Email: weberw@mskcc.org

**Supporting figures**


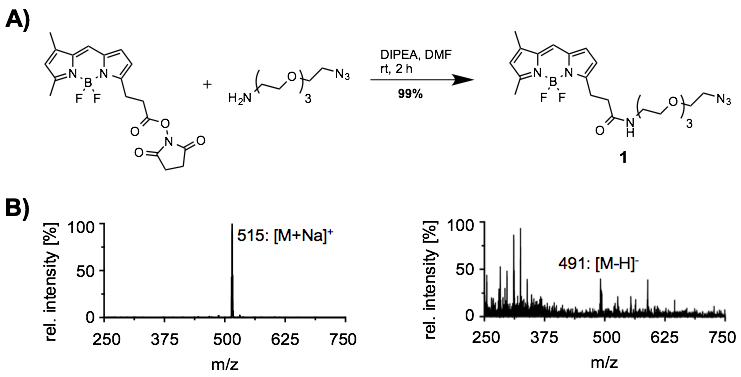


Figure S1: Synthesis and analytical data of BODIPY-azide **1**. (A) Amine-reactive BODIPY-Fl NHS ester was coupled over 2 h to 11-Azido-3,6,9-trioxaundecan-1-amine to obtain BODIPY-azide **1** in 99% yield.
(B) Positive and negative-polarized ESI-MS of BODIPY-azide **1**.

Figure S2: Synthesis of BODIPY-BBN **5** using CuAAC click chemistry.


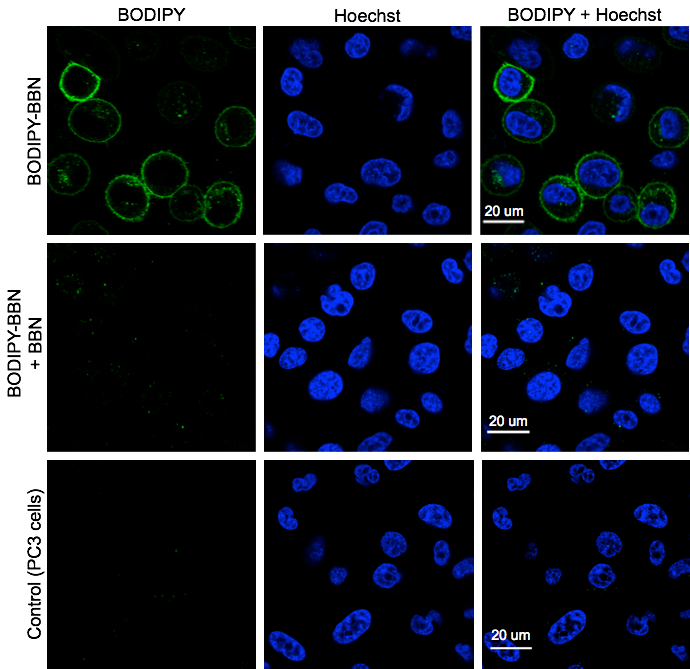


Figure S3: In vitro cell binding study of BODIPY-BBN **5** using GRPr overexpressing PC-3 cells. BODIPY-BBN (green) and blue nuclear cell stain (blue).


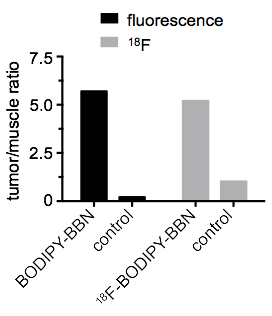


Figure S4: Quantification of tumor uptake comparing fluorescent imaging as well as gamma-counting.


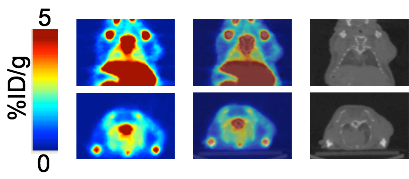


Figure S5: *In vivo* small animal PET/CT images (60 min) of PC-3 tumor bearing nude mice after intravenous injection of ^18^F-BODIPY-BBN **3** (55 ± 10 µCi) in PBS (4% DMSO, 200 µL).


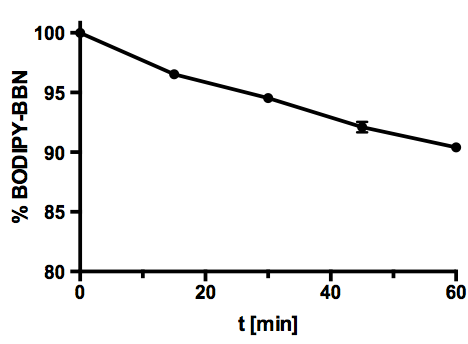


Figure S6: Percent intact fluorescent ^19^F-BODIPY-BBN in human serum at different time points (0, 15, 30, 45, and 60 min) after incubation at 37 °C.

**Chemistry and analytical data**

**Preparation of alkyne-modified bombesin analog 4.**

The alkyne-modified peptide, bombesin analog RM2 [1] (BBN, Leu-Sta-His-Gly-Val-Ala-Trp-Gln-D-Phe-NH_2_) was synthesized manually according to standard Fmoc chemistry [2] using Rink amide MBHA resin and Fmoc-protected amino acids [1]. The spacer and the 4-pentynoic acid were attached to the peptide sequence with HATU as an activating agent. A solution of TFA, anisole, and water (1.0 mL, ratio of 95:3:2) was added to the alkyne-modified bombesin (100 mg) and stirred at room temperature for 4 h. Afterwards diethyl ether (5.0 mL) was added to precipitate the peptide. After centrifugation the supernatant was removed and the peptide was dissolved in water (1.8 mL) and acetonitrile (0.2 mL). Purification by HPLC (10 mL/min, 5% to 95% B in 20 min) afforded the alkyne-modified bombesin **4** (18 mg, 71%) as a white solid: t_R_ = 10.5 min. ESI-MS(+): *m/z* (%) = 1334.72 (60) [M+H]^+^, 667.64 (100) [M+2H]^2+^. HRMS (ESI): *m/z* calcd for C_67_H_97_N_16_O_13_: 1333.7421; found: 1333.7367 [M+H]^+^_._

**References**

1. Mansi, R. et al. Development of a potent DOTA-conjugated bombesin antagonist for targeting GRPr-positive tumours. Eur J Nucl Med Mol Imaging 2011;38,97-107.

2. Atherton, E.; Sheppard, R. Fluorenylmethoxycarbonyl-polyamide solid phase peptide synthesis. General principles and development. Oxford: Oxford Information Press 1989
